# CT Findings of Asthma and Asthma-Mimicking Conditions

**DOI:** 10.7759/cureus.108457

**Published:** 2026-05-07

**Authors:** Abdullah H Al-Sergani, Renad H Zaini, Abdulrahman H Alsergani, Fahad A Alfaiz, Abdullah AlMeaither, Mnahi B Saeedan

**Affiliations:** 1 Radiology, University of Texas Health Science Center at Houston, Houston, USA; 2 Medical Education, Princess Nourah Bint Abdulrahman University, Riyadh, SAU; 3 Medical Education, King Saud University, Riyadh, SAU; 4 Radiology, McGill University, Montreal, CAN; 5 Radiology, King Saud University, Riyadh, SAU; 6 Cardiothoracic Radiology, King Faisal Specialist Hospital and Research Centre, Riyadh, SAU

**Keywords:** ct-high resolution, ct-quantitative, lung, mediastinum, thorax

## Abstract

Asthma is a disorder that requires imaging to assess disease severity, complications, and atypical presentations. High-resolution computed tomography (HRCT) has become increasingly valuable in identifying imaging features of asthma and structural mimickers. Asthma typically demonstrates bronchial wall thickening, mosaic attenuation, and air trapping. Chronic eosinophilic pneumonia is characterized by peripheral ground-glass opacities and consolidations. Allergic bronchopulmonary aspergillosis presents with central bronchiectasis, mucus plugging, and high-attenuation mucus. Eosinophilic granulomatosis with polyangiitis reveals nonspecific findings. Excessive central airway collapse is characterized by dynamic tracheal narrowing. Diffuse idiopathic pulmonary neuroendocrine cell hyperplasia is marked by multiple small nodules and mosaic attenuation. Recognizing the differences between the aforementioned entities may decrease time to diagnosis and subsequently time to treatment.

## Introduction and background

Asthma affects an estimated 262 million people globally [1] and is defined by chronic inflammation of the lower respiratory tract [2]. It presents with respiratory symptoms, variable airflow limitation, and, in some cases, chronic airflow obstruction [3]. Diagnosis requires symptoms such as cough or dyspnea, along with objective evidence of reversible airway obstruction [4]. Up to one-third of adults may be over-diagnosed due to asthma's non-specific and variable presentation [5]. This underscores the importance of distinguishing true asthma from mimickers and recognizing asthma-associated conditions, particularly in severe or treatment-resistant cases [6].

Although not routinely used for diagnosis, imaging helps exclude complications such as pneumonia or pneumothorax, typically via chest radiography. Advances in high-resolution computed tomography (HRCT), including thin-section imaging and high-frequency reconstruction, have enhanced airway visualization and renewed interest in imaging's diagnostic role. Despite its utility, asthma shares CT features such as bronchial wall thickening and air trapping with other conditions, making differentiation challenging. Subtle differences, especially in the frequency and distribution of findings, can aid diagnosis. HRCT also plays a role in monitoring treatment response in atypical cases [7-9].

This narrative review outlines the CT imaging features of asthma, asthma-associated conditions, and asthma mimickers (Table 1). Associated conditions include chronic eosinophilic pneumonia (CEP), allergic bronchopulmonary aspergillosis (ABPA), and eosinophilic granulomatosis with polyangiitis (EGPA). Asthma mimickers include expiratory central airway collapse (ECAC) and diffuse idiopathic pulmonary neuroendocrine cell hyperplasia (DIPNECH). Emphasizing CT-based distinctions among these entities may enhance diagnostic accuracy and guide clinical management.

**Table 1:** Summary of key findings and unique CT characteristic of each pathology. CEP: Chronic eosinophilic pneumonia; ABPA: Allergic bronchopulmonary aspergillosis; EGPA: Eosinophilic granulomatosis with polyangiitis; ECAC: Expiratory central airway collapse; EDAC: Excessive dynamic airway collapse; TBM: Tracheobronchomalacia; COPD: Chronic obstructive pulmonary disease; DIPNECH: Diffuse idiopathic pulmonary neuroendocrine cell hyperplasia

Disease	CT findings	Clinical notes
Asthma	CT can appear normal. Abnormal findings include bronchial wall thickening, mucus plugs, mosaic attenuation (inspiratory), and air trapping (expiratory)	Chronic cough, dyspnea, wheezing, reversible airflow obstruction. Diagnosis: Clinical (spirometry), but CT aids in severe/refractory cases
CEP	Peripheral ground-glass opacities and consolidations, which could be fleeting	Often in asthmatics with atopy (e.g., eczema). Labs: Peripheral eosinophilia; exclusion of other eosinophilic causes (e.g., ABPA, EGPA)
ABPA	Central bronchiectasis, mucus plugging ("finger-in-glove" appearance), high-attenuation mucus centrilobular nodules, and bronchial wall thickening	Poorly controlled asthma, productive cough, linked to Aspergillus hypersensitivity. Labs: Elevated serum IgE, *A. fumigatus*-specific IgE/IgG; eosinophilia (>500 cells/μL)
EGPA	Air trapping, bronchial wall thickening, ground-glass and consolidative opacities, pulmonary embolism, paranasal sinus involvement	Asthma, eosinophilia, vasculitis, multi-organ involvement (e.g., neuropathy, purpura). Labs: Peripheral eosinophilia; ANCA-positive (50%)
ECAC	Expiratory dynamic collapse: ≥70% reduction in airway caliber EDAC: Posterior wall bowing with crescent-shaped lumen on expiratory CT TBM: Cartilage deformity with lunate-shaped on inspiratory CT and flattening cartilage on expiratory CT	Symptoms: Chronic cough, dyspnea; often misdiagnosed as asthma/COPD. Associations: Severe asthma in older women, COPD
DIPNECH	Multiple predominantly small nodules (<5 mm, carcinoid tumorlet, precursor to carcinoid tumors), bronchial wall thickening, inspiratory mosaic attenuation, and expiratory air trapping	Symptoms: Chronic cough, dyspnea; mimics asthma but refractory to treatment, in middle-aged/older women

Methodology and limitations

This review was conducted in late 2025 to early 2026. It involved studies ranging from 1992 to 2026. A greater concentration of studies was between the years 2020 and 2024, comprising approximately 40% (23) of the total referenced studies. No strict inclusion or exclusion criteria were used, given that this was a narrative review. Radiology-focused journals were prioritized, where 22% (13) of all references were sourced from.

All figures used in this review were encountered during clinical practice by the authors and subsequently collected from multiple institutions in line with each institutional policy. Adequate anonymization was performed to remove patient-identifying information.

Although this review provides a comprehensive overview of CT findings of asthma and asthma mimickers, it has several limitations. Notably, the search strategy was not as stratified and rigid as a systematic review. Another limitation is that the number of references is naturally limited for each pathology, given the wide scope of this review.

## Review

Asthma

Asthma is a chronic, multifactorial disease characterized by airway remodeling and subsequent bronchial narrowing. This remodeling is driven by bronchoconstrictive mediators, such as histamines, leukotrienes, and prostaglandins, although the upstream triggers for their release remain unclear [10]. While asthma is primarily a clinical diagnosis, CT has demonstrated value in assessing the extent of airway remodeling, particularly in severe or treatment-resistant cases. CT-derived airway wall thickness correlates strongly with histopathologic measurements from endobronchial biopsies [11], and increased wall thickness has been associated with lower forced expiratory volume in one second (FEV₁) and a higher risk of exacerbations [12].

Imaging Features

The hallmark CT features of asthma include bronchial wall thickening, with inspiratory mosaic attenuation and expiratory air trapping. Reported frequencies vary: Khadadah et al. found air trapping in 78% and wall thickening in 57% of patients [13], whereas Lynch et al. reported wall thickening in 92% but air trapping in only 21% [14]. Paganin et al. observed bronchial wall thickening in up to 16% of patients, depending on disease severity [15]. These discrepancies may reflect selection bias, as imaging is more commonly performed in severe cases where CT abnormalities are more pronounced. Less frequent findings include mucus plugging, reported in 18%-30% of patients [13,15], and bronchiectasis, with prevalence varying by severity [14].

Chronic eosinophilic pneumonia (CEP)

Eosinophilic pneumonia refers to eosinophilic infiltration of the lung parenchyma, identified by the presence of eosinophils in lung tissue, bronchoalveolar lavage fluid, and often peripheral blood. It is classified into acute eosinophilic pneumonia (AEP) and CEP [16]. Clinically, AEP presents abruptly - typically within two weeks of exposure to an inciting antigen - often associated with new or altered smoking habits and usually without peripheral eosinophilia. In contrast, CEP has a more insidious onset, with symptoms such as progressive dyspnea and wheezing developing over months. Patients with CEP frequently have a history of asthma, atopic conditions (including eczema, nasal polyposis, and urticaria), and peripheral eosinophilia [9,17,18]. Diagnosis of CEP requires a combination of clinical features, peripheral eosinophilia, characteristic CT findings, and exclusion of other causes of eosinophilic lung disease (e.g., tropical eosinophilia, illicit drug use, allergic bronchopulmonary aspergillosis (ABPA), eosinophilic granulomatosis with polyangiitis (EGPA), and hyper-eosinophilic syndrome) [9].

Imaging Features

**Figure 1:**
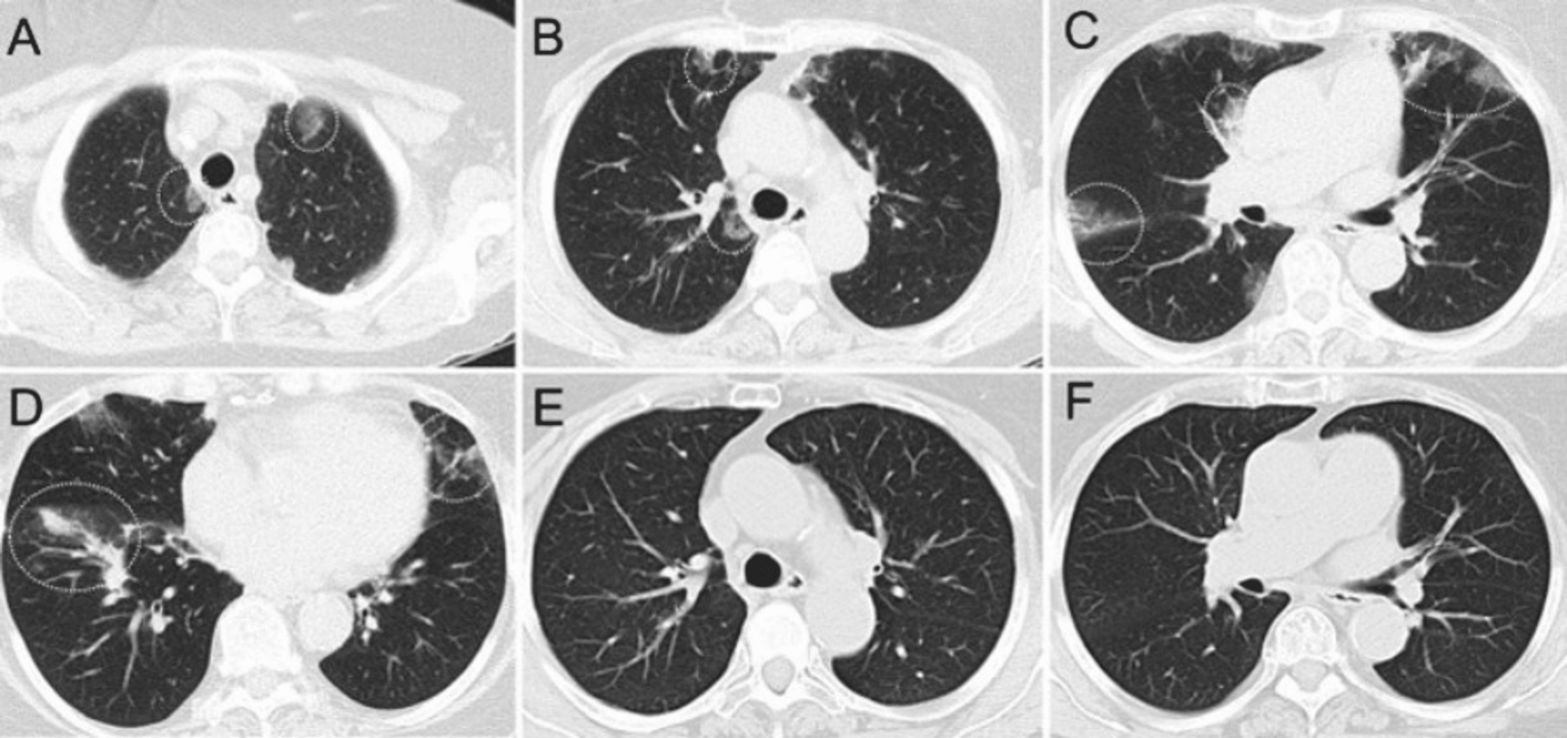
Chronic eosinophilic pneumonia in a patient with a history of asthma and peripheral eosinophilia. Axial CT images (A-D) show bilateral, multifocal, peripheral ground-glass opacities (some areas annotated with dotted circles) associated with reticular opacities and small consolidations. Axial images from a follow-up CT (E and F), after treatment with steroids, demonstrate interval resolution of lung opacities.

**Figure 2:**
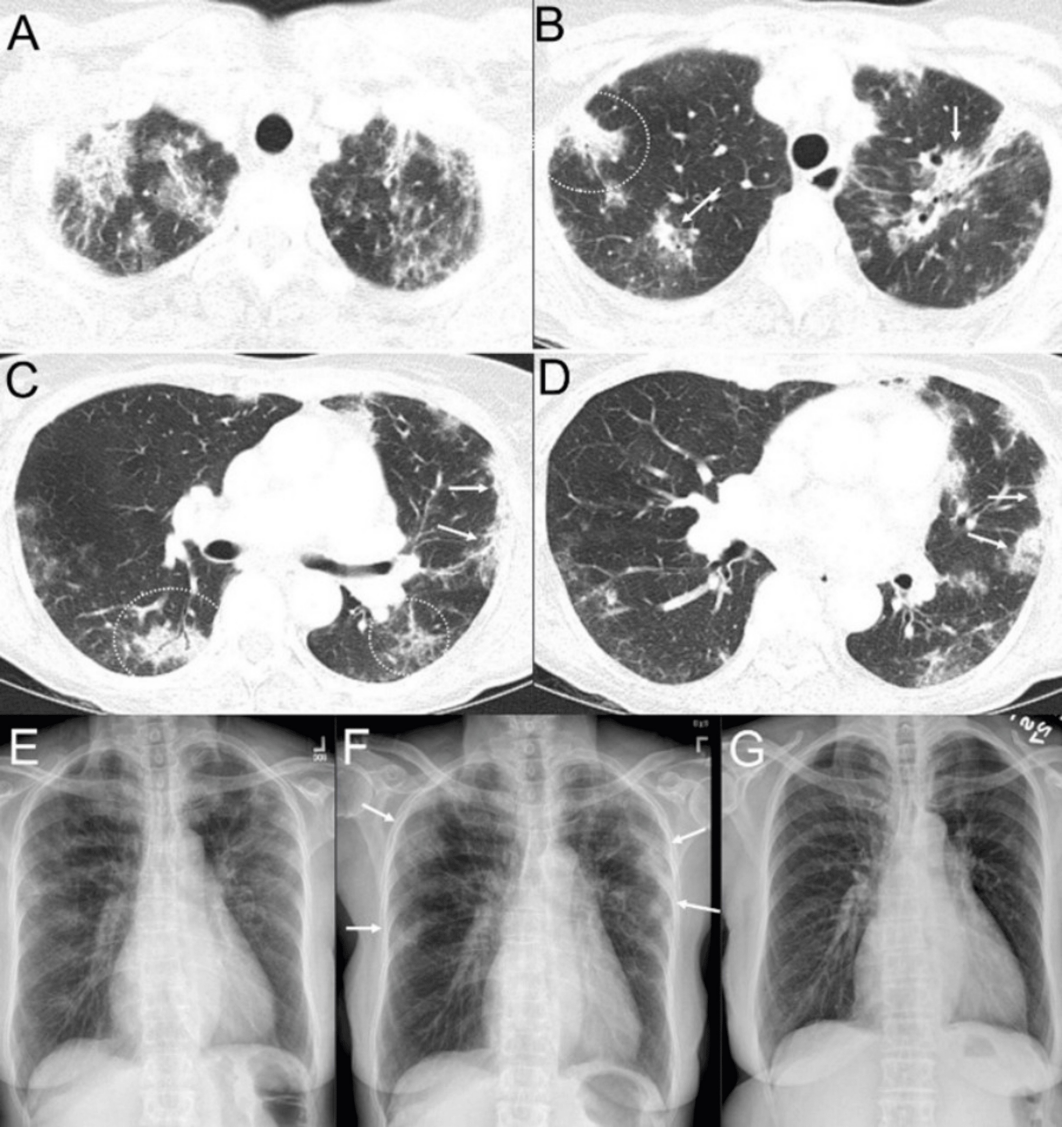
Chronic eosinophilic pneumonia in a patient presenting with chronic cough, wheezing, worsening radiographic lung opacities, and peripheral eosinophilia. Axial CT images (A-D) show bilateral, multifocal upper lobe predominant and peripheral to a lesser extent peribronchial mixed ground-glass opacities and consolidations associated with mild reticular opacities (some areas annotated with arrows and dotted circles). A frontal chest radiograph obtained one month prior to the CT (E) shows upper lobe and peripheral predominant hazy lung opacities with small consolidations. A frontal chest radiograph obtained a few days prior to the CT (F) shows persistent and increased lung opacities (arrows). The patient underwent bronchoalveolar lavage, which revealed an eosinophil count of 25%. A frontal chest radiograph obtained a few months after the CT (G), following steroid treatment, shows resolution of lung opacities.

The hallmark imaging features of CEP include airspace consolidations and ground-glass opacities. Johkoh et al. reported consolidations in 100% and ground-glass opacities in 88% of patients, with a predominantly peripheral distribution in 85%; none showed central predominance, as shown in Figures 1-2. Lobar involvement was random in 69% of cases [19]. Mehrian et al. similarly observed ground-glass opacities in 89% and consolidations in 45%, with peripheral involvement in 70% [20]. Other findings include reticulations - indicative of interlobular septal thickening and centrilobular nodules, both typically seen in peripheral lung regions. Interlobular septal thickening is reported in 18-62% of cases, and centrilobular nodules in 14-38% [19-21].

Allergic bronchopulmonary aspergillosis (ABPA)

*Aspergillus *lung disease is primarily determined by the host's immune status. Invasive forms - such as angioinvasive pulmonary aspergillosis and tracheobronchial disease - typically occur in immunocompromised patients. In contrast, non-invasive forms, seen in immunocompetent individuals, include aspergilloma and ABPA [22].

ABPA is a hypersensitivity reaction to inhaled *Aspergillus *antigens, predominantly affecting individuals with asthma or cystic fibrosis. In atopic patients, exposure elicits an exaggerated IgE-mediated response, in contrast to the IgG/IgA-mediated clearance seen in healthy individuals [23]. Clinically, ABPA presents with poorly controlled asthma, recurrent wheezing, and a productive cough. Diagnosis relies on the integration of clinical, serological, and radiological findings, with chest CT playing a key role [24].

The diagnostic criteria for ABPA, established by the International Society for Human and Animal Mycology, require the presence of a predisposing condition, such as asthma or cystic fibrosis, along with either immediate skin reactivity to *Aspergillus *antigen or elevated serum-specific IgE to *Aspergillus fumigatus*, and elevated total IgE levels. In addition, at least two of the following minor criteria must be met: positive *A. fumigatus*-specific IgG (precipitating antibodies), radiologic findings consistent with ABPA, or peripheral eosinophilia exceeding 500 cells/μL in patients not on corticosteroids [25-27]. Diagnosis is frequently delayed or overlooked, which can postpone treatment and result in progressive airway and parenchymal damage, including the development of bronchiectasis [28].

Imaging Features

**Figure 3:**
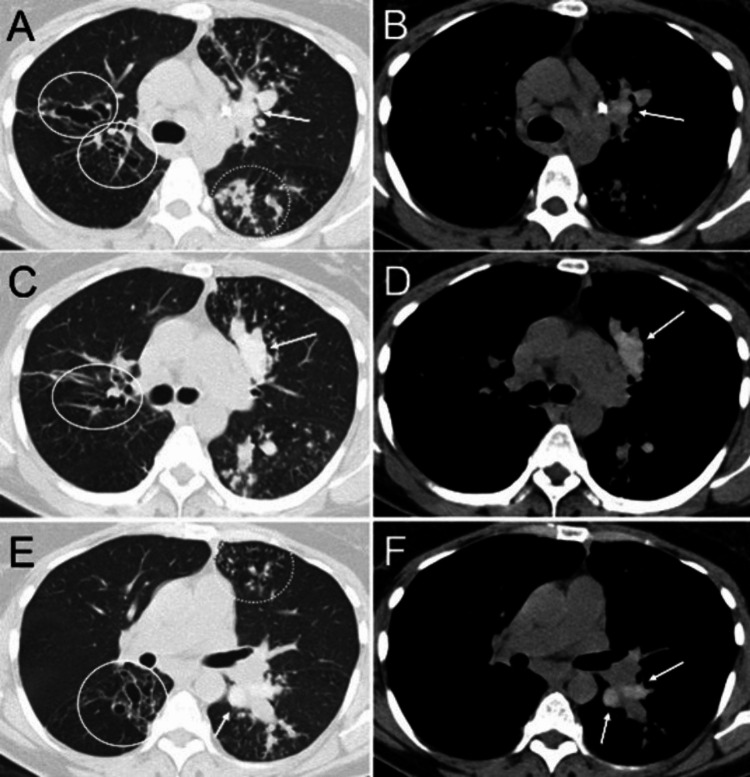
Allergic bronchopulmonary aspergillosis in a patient with asthma, environmental allergies, chronic persistent cough, and positive skin prick test for Aspergillus. Axial CT images using lung window settings (A, C, E) and mediastinal window settings (B, D, F) demonstrate central and upper lobe predominant bronchiectasis (solid circles), high-attenuation mucus plugs (arrows), and centrilobular nodules with a tree-in-bud pattern (dashed circles).

The hallmark CT finding in ABPA is the presence of bronchoceles-dilated bronchi filled with mucus - typically demonstrating a branching "finger-in-glove" appearance [23]. Another important feature is high-attenuation mucus (HAM), defined as mucus denser than adjacent skeletal muscle [29]. Kaur et al. [30] reported centrilobular nodules in 86% of patients, bronchiectasis in 78% (with both central and peripheral distribution), and bronchoceles in 59%, of which 61% demonstrated HAM, which is well demonstrated in Figure 3. Agarwal et al. [31] found central bronchiectasis in 63% of cases, with peripheral extension in 43%, but no instances of isolated peripheral bronchiectasis. Mucoid impaction was present in 50% of patients, predominantly hypodense, while centrilobular nodules were seen in 47%. In a separate study evaluating steroid response in ABPA, bronchiectasis was observed in 92% of patients, followed by bronchial wall thickening (89%), centrilobular micronodules (82%), and mucoid impaction (61%), of which only 36% exhibited high attenuation [32]. Variability in HAM detection may reflect subjective interpretation and differences in CT windowing and display settings.

Eosinophilic granulomatosis with polyangiitis

Eosinophilic granulomatosis with polyangiitis is a small-vessel necrotizing vasculitis within the antineutrophil cytoplasmic antibody (ANCA)-associated vasculitis group, alongside granulomatosis with polyangiitis and microscopic polyangiitis [33]. Approximately 50% of EGPA patients are ANCA-positive, predominantly for p-ANCA/anti-myeloperoxidase [34]. EGPA is distinguished by its strong association with asthma and peripheral eosinophilia, with eosinophilic infiltrates affecting multiple organs [35]. It presents across three clinical phases: the atopic phase (asthma, chronic rhinosinusitis), eosinophilic phase (marked eosinophilia and early organ involvement), and vasculitic phase (multisystem small-vessel vasculitis) [36].

Manifestations vary and may include pulmonary symptoms (asthma, sinusitis), myocarditis, peripheral neuropathy, purpura, scleritis, alveolar hemorrhage, and glomerulonephritis [37]. Due to its heterogeneity, no standardized diagnostic criteria exist. Diagnosis remains primarily clinical, supported by eosinophilia and ANCA testing. HRCT has limited diagnostic value due to nonspecific findings, but it is useful for assessing disease severity and monitoring relapses [36]. EGPA is associated with a high risk of acute arterial and venous thromboembolic events, including deep vein thrombosis and pulmonary embolism [34].

Imaging Features

**Figure 4:**
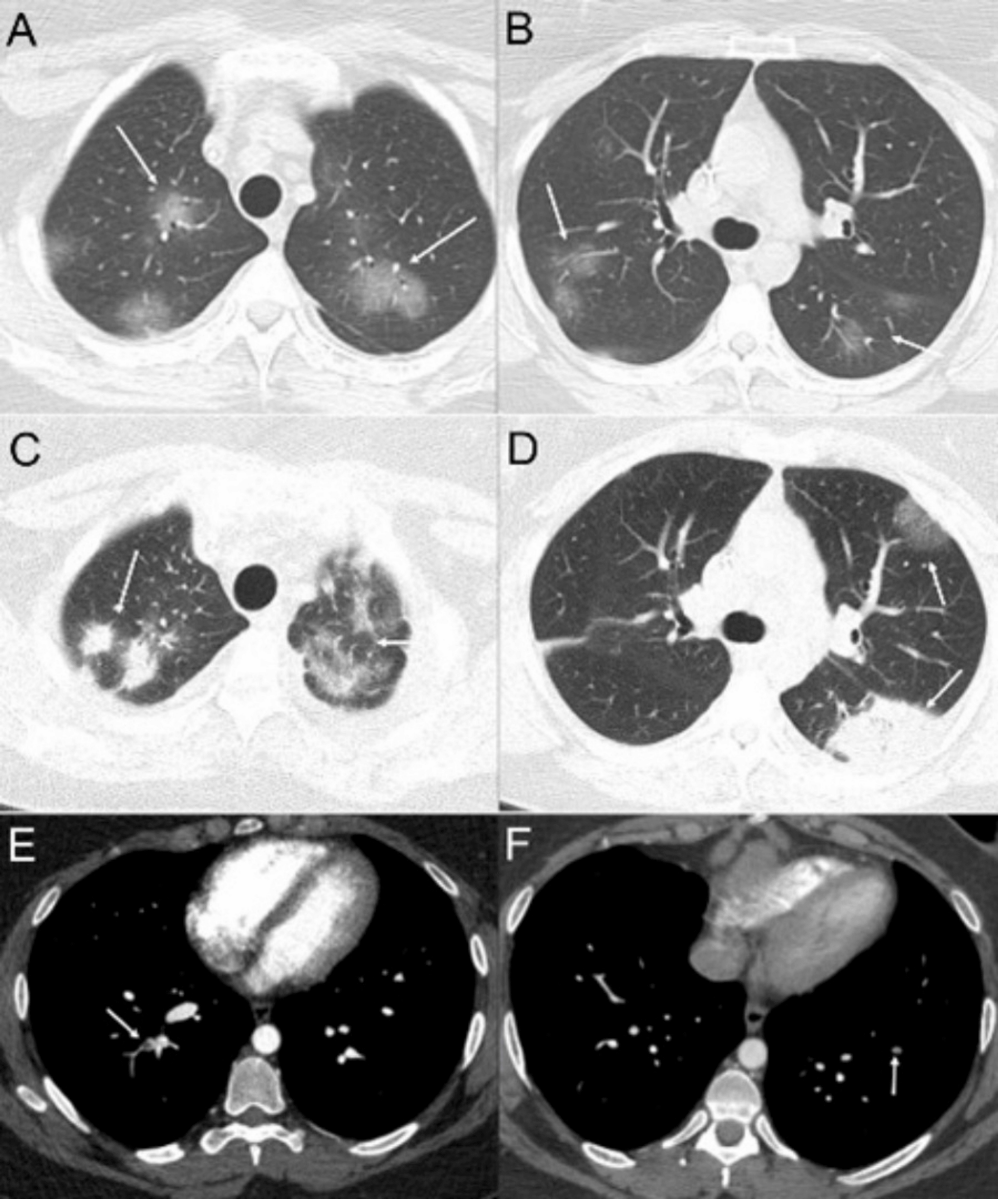
Eosinophilic granulomatosis with polyangiitis, diagnosed by a history of asthma, peripheral eosinophilia, paranasal sinus disease, and cutaneous vasculitis. Axial CT images (A and B) demonstrate bilateral, multifocal ground-glass opacities (arrows). These opacities are resolved on a subsequent follow-up CT. Axial images from a follow-up CT performed several years after the initial scan (C and D) reveal new multifocal ground-glass opacities and consolidations (arrows). Additionally, axial CT images from two different pulmonary CT angiography exams (E and F) show filling defects in the pulmonary arteries consistent with pulmonary emboli (arrows).

Although CT findings in EGPA are non-specific, they aid in evaluating disease burden and progression [37]. Lin et al. [38] reported frequent air-trapping (98%), bronchial wall thickening (97%), and randomly distributed ground-glass opacities (74%), with tree-in-bud nodules in 63%. Delestre et al. found bronchial wall thickening (70%) most common, followed by air-trapping (63%) and ground‑glass opacities (33%), noting that persistent ground‑glass opacities were linked to poorer outcomes [39]. Choi et al. identified ground‑glass opacities (100%), nodules (89%), and consolidations (56%) as prevalent findings [40]. Paranasal sinus involvement, affecting roughly 50% of patients, typically presents as rhinitis, polyposis, or sinusitis [41]. Examples of EGPA CT findings are found in Figure 4.

Miscellaneous

This section discusses asthma mimickers without intrinsic eosinophilic pathology, including ECAC and DIPNECH. CT imaging plays a key role in the detection of these distinct clinical entities [42,43].

Excessive central airway collapse includes tracheobronchomalacia (TBM) and EDAC, both defined by exaggerated narrowing of the tracheobronchial tree during expiration [44]. TBM results from weakening of the anterolateral cartilage, whereas EDAC is due to laxity of the posterior membranous wall from impaired longitudinal muscle fibers. Both conditions may present with cough and dyspnea and are frequently misdiagnosed as asthma or coexist with it [45]. EDAC is commonly associated with obstructive lung diseases, such as asthma and chronic obstructive pulmonary disease (COPD) [46].

EDAC is characterized by inward bowing of the posterior membranous wall with preserved cartilaginous rings, while TBM involves structural abnormalities of the cartilage, affecting the trachea (tracheomalacia), bronchi (bronchomalacia), or both (TBM) [47]. EDAC is more prevalent than TBM, especially among older women and patients with severe asthma; one severe asthma cohort reported EDAC and TBM in 70% and 19% of patients, respectively [48].

Flexible bronchoscopy remains the gold standard for diagnosis, providing real-time assessment of dynamic airway collapse during various respiratory maneuvers [49]. CT performed during inspiratory and dynamic expiratory phases offers a non-invasive alternative, with a ≥70% reduction in airway caliber currently considered the most specific diagnostic threshold [47]. The traditionally used ≥50% cutoff lacks specificity, as 78% of healthy individuals met this criterion in a study by Boiselle et al. [50].

**Figure 5:**
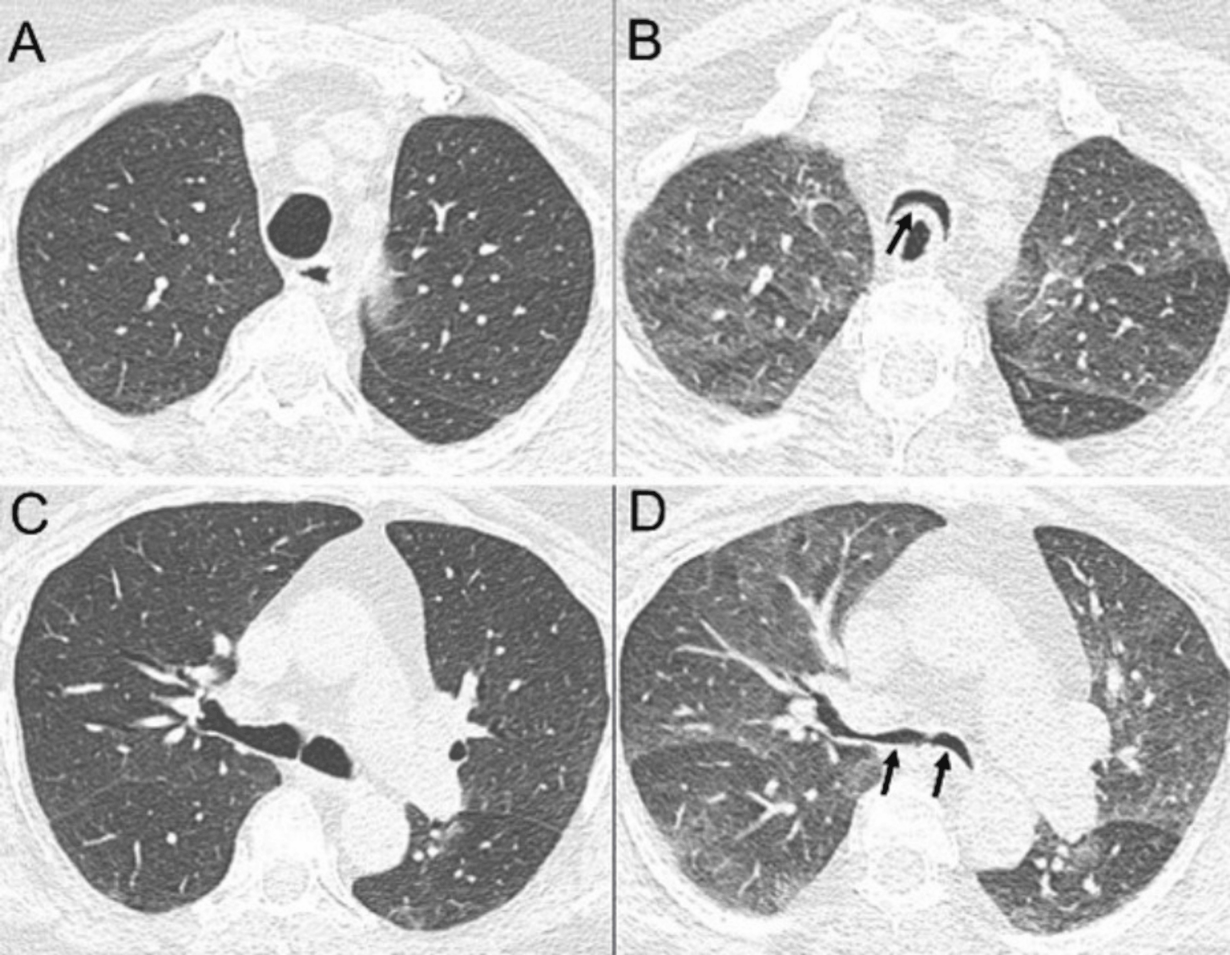
Excessive dynamic airway collapse. Axial CT images during inspiration (A and C) and expiration (B and D) demonstrate excessive dynamic collapse of the trachea and main bronchi (arrows), along with patchy expiratory air trapping. Notably, there is excessive anterior bowing of the posterior tracheal wall during expiration resulting in a crescent-shaped trachea.

**Figure 6:**
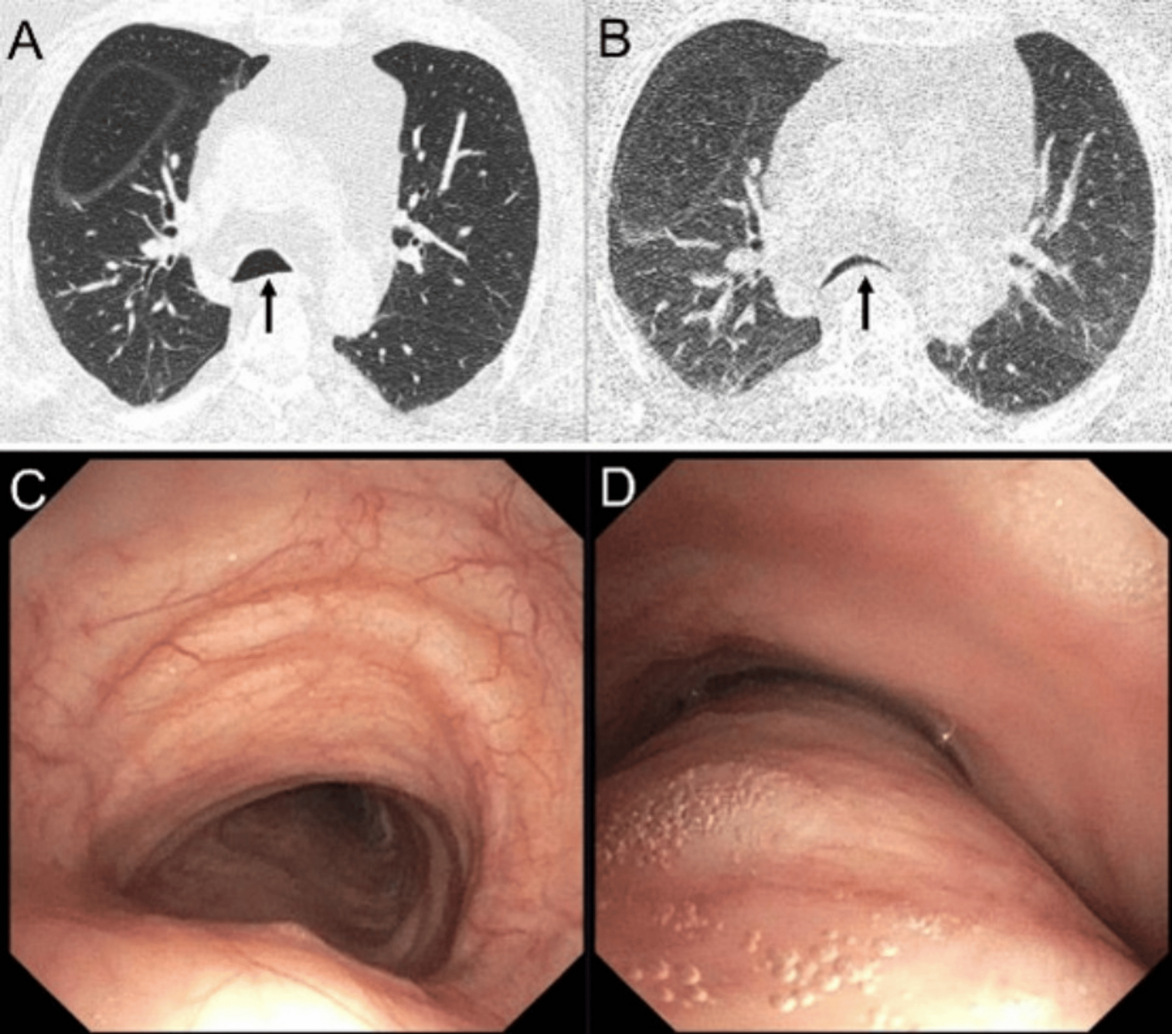
Tracheomalacia. Axial CT images during inspiration (A) and expiration (B) show a flattened tracheal morphology during both phases, with more than 70% reduction in anterior-posterior diameter during expiration (arrows) and patchy expiratory air trapping. Bronchoscopy images during inspiration (C) and expiration (D) reveal tracheal collapse during expiration.

On dynamic expiratory CT, exaggerated bowing of the posterior membrane with preserved integrity of the tracheal cartilage forms a crescent-shaped lumen in EDAC, as shown in Figure 5. In comparison, TBM demonstrates collapse of the anterior and lateral walls results in flattened tracheal cartilage [47], as shown in Figure 6. TBM may be suspected on standard chest CT or inspiratory images. Cartilage softening alters the normal tracheal index (coronal-to-sagittal diameter ratio, normally around 1), with anterior wall weakness producing a lunate shape and an increased index [47].

Diffuse idiopathic pulmonary neuroendocrine cell (PNEC) hyperplasia is an uncommon but increasingly identified pulmonary condition that predominantly affects middle-aged to older women. It involves diffuse proliferation of PNECs and is regarded as a precursor to carcinoid tumorlets and tumors. Clinically, DIPNECH may be accompanied by constrictive bronchiolitis, often presenting with chronic cough, dyspnea, and obstructive spirometry patterns that frequently lead to misdiagnosis as asthma. On chest CT, the hallmark findings are multiple small, noncalcified pulmonary nodules accompanied by mosaic attenuation, reflecting underlying obstructive small airways disease [51-55].

**Figure 7:**
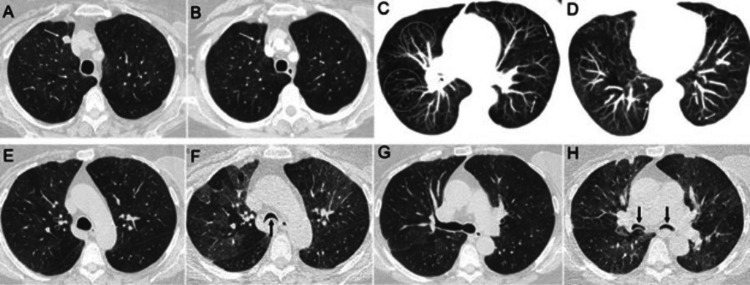
Diffuse idiopathic pulmonary neuroendocrine cell hyperplasia with a carcinoid tumor. Axial CT image (A) and a corresponding axial image from a CT performed nine years earlier (B) demonstrate an indolent interval enlargement of a right upper lobe nodule (arrows). Axial maximum intensity projection images (C and D) reveal numerous small lung nodules (<6 mm), some marked by arrows or dashed circles; most of these nodules remained stable over several years. During inspiration scans (E and G), mosaic attenuation is evident, while expiration scans (F and H) show patchy expiratory air trapping along with excessive dynamic airway collapse of the trachea and main bronchi (arrows). Surgical resection confirmed the presence of a carcinoid tumor in the right upper lobe nodule, along with adjacent carcinoid tumorlets.

Little et al. described key CT features in a series of DIPNECH cases, where all patients demonstrated centrilobular nodules - most commonly in the lower lobes (75%) and peripherally distributed (56%) - along with mosaic attenuation from air trapping in 91% of patients [55]. Additionally, bronchial or bronchiolar wall thickening was observed in 75% of cases, and 74% of lobes had more than 10 nodules smaller than 5 mm, underscoring the diffuse nature of the disease. These findings were corroborated by Chung et al., who similarly reported mosaic attenuation and centrilobular nodules in all cases (100%), with over 10 small nodules per lobe in 74% of patients [56], as demonstrated in Figure 7. Recognizing this specific radiologic pattern is critical for differentiating DIPNECH from more common obstructive diseases such as asthma or chronic bronchitis, enabling earlier and more accurate diagnosis.

CT protocol

The standard protocol includes a non-contrast HRCT with inspiratory and low-dose dynamic expiratory phases. The inspiratory scan, acquired at end inspiration, assesses bronchial wall thickening, mucus plugging, mosaic attenuation, and parenchymal abnormalities. The dynamic expiratory scan is essential for detecting air trapping-a hallmark of small-airway obstruction [57], as well as ECAC, which can mimic or coexist with asthma [47]. Although intravenous contrast is not routinely required, it may be administered when vascular pathology is clinically suspected (e.g., pulmonary embolism).

Traditional CT measures of airway morphometry and air trapping primarily detect severe asthma. In contrast, quantitative CT assessments of lung expansion - capturing its magnitude, direction, and variability - differentiate more effectively across all severity levels, revealing distinct mechanical patterns among asthma subtypes. These functional imaging techniques offer a more nuanced understanding of disease heterogeneity and may help identify patients unlikely to respond to standard biologic therapies. Continued development and clinical integration of these methods could enhance monitoring of disease progression and treatment response, particularly in severe asthma [58].

## Conclusions

Asthma is a heterogeneous disease with imaging features that often overlap with eosinophilic lung diseases and structural airway disorders. While not central to initial diagnosis, HRCT plays a crucial role in identifying associated conditions, such as CEP, ABPA, and EGPA, as well as mimickers, such as ECAC and DIPNECH - particularly in atypical or treatment-refractory cases. Recognizing characteristic imaging patterns can refine the differential diagnosis, prevent misclassification, and support more accurate clinical decision-making in complex asthma presentations.
